# Caring for a Family Member Living With Dementia When Adult Day Services Close

**DOI:** 10.1093/geroni/igab046.421

**Published:** 2021-12-17

**Authors:** Katherine Marx, Lauren Parker, Joseph Gaugler, Holly Dabelko-Schoeny, Laura Gitlin

**Affiliations:** 1 Johns Hopkins School of Nursing, Baltimore, Maryland, United States; 2 Johns Hopkins Bloomberg School of Public Health, Baltimore, Maryland, United States; 3 University of Minnesota, Minneapolis, Minnesota, United States; 4 The Ohio State University, The Ohio State University, Ohio, United States; 5 Drexel University, College of Nursing and Health Professions, Drexel University, Pennsylvania, United States

## Abstract

Adult Day Service (ADS) centers play an important role in community services that help families keep a person living with dementia (PLWD) at home. We interviewed 33 family caregivers about their experience during the COVID-19 Pandemic and the shutdown of the ADS centers where the PLWD attends. All 33 (100%) reported that the ADS center was shut for a period of time (range: 2 weeks – remain closed). Caregivers reported a decline in their physical health (33%,n=11) and mental health (52%,n=17) and an increase in feelings of loneliness (48%,n=16). For the PLWD, the caregivers noted, a decline in physical (48%,n=16) and mental (55%,n=18) health and an increase in behaviors (39%,n=13). The shutdown of most ADS centers across the country due to the COVID-19 pandemic has had implications not only for the ADS sites but for the families that entrust them with the care for a family member.

